# The Efficacy and Safety of Chinese Herbal Decoction in Type 2 Diabetes: A 5-Year Retrospective Study

**DOI:** 10.1155/2016/5473015

**Published:** 2016-08-30

**Authors:** Jiaxing Tian, Fengmei Lian, Xiaotong Yu, Yashan Cui, Tianyu Zhao, Yang Cao, Xiaolin Tong

**Affiliations:** ^1^Guang'anmen Hospital, China Academy of Chinese Medical Sciences, Beijing 100053, China; ^2^Beijing University of Chinese Medicine, Beijing 100029, China; ^3^Xiyuan Hospital, China Academy of Chinese Medical Sciences, Beijing 100091, China; ^4^First Teaching Hospital, Tianjin University of Traditional Chinese Medicine, Tianjin 300192, China

## Abstract

*Background*. The study was designed to assess the efficacy and safety of Chinese herbal decoction in treating outpatients with T2DM.* Methods*. All patients enrolled received decoction for at least 6 months. The primary outcome was the control rate of HbA1c and the change in HbA1c. FPG, 2hPG, HOMA-IR, and HOMA-*β* were also collected and evaluated.* Results*. The control rates after treatment at months 6, 12, 18, 24, 36, 48, and 60 were 45.07%, 52.78%, 47.22%, 45.83%, 50.00%, 57.14%, and 40.00%. Multiple linear regression showed the change of HbA1c has a significant relationship with the baseline HbA1c and duration of DM and BMI (*p* < 0.05). Both FPG and 2hPG levels significantly decreased compared to the baseline (*p* < 0.05). Chinese herbal decoction also improved islet cell function with decreased HOMA-IR and increased HOMA-*β* (*p* < 0.05). 19 and 4 subjects deactivated the antidiabetes drugs or insulin, respectively, after taking decoction. One subject developed DKD and one developed DPN, and another subject showed abnormal liver function which was irrelevant to decoction treatment.* Conclusions*. Chinese herbal decoction significantly enhanced the hypoglycemic action and had certain effect on protecting islet cell function. As a candidate diabetes therapy, it may reduce the use of antidiabetes drugs and slow the progression to diabetes complications.

## 1. Introduction

Diabetes mellitus (DM) has become an important public health problem worldwide [1, 2]. A recent global study indicated that the prevalence of DM was rising rapidly, particularly in developing countries [3]. With the rapid economic development, elevated standard of living, dietary shifts, lifestyle alterations, and aging, China has the largest number of diabetic patients in the world [4]. Currently the overall prevalence of diabetes was estimated to be 11.6% and the prediabetes was estimated to be 50.1% in Chinese adults [4]. It has been estimated that a total of 12.9 million people died from ischemic heart disease and stroke in the world, while diabetes is the main risk factor [5, 6]. Studies have shown that good glycemic control is critical for patients with type 2 diabetes mellitus (T2DM), since the HbA1c level is correlated with diabetic complications [7, 8]. However, according to the latest survey among 22.31 million Chinese T2DM patients, it was shocking that more than two-thirds could not effectively control their HbA1c levels [9]. Therefore, it is urging to expand the treatment.

Chinese herbal medicine has more than 2000 years of medical practical history. It is an excellent resource for discovering new innovative medications. Previous studies have reported the efficacy and safety of several Chinese herbal medicines that effectively reduced blood glucose and HbA1c levels in diabetic and prediabetic patients [10–13]. Thus, we believe that Chinese herbal medicine may play a role in treating this very common metabolic disease.

Chinese herbal decoction has been widely used in China for diabetes management. However, there is a lack of convincing clinical evidence of glycemic control treated by herbal decoction, especially for changes in islet cell function. Additionally, the change in dosage of antidiabetes drugs or insulin after combining herbal decoction is rarely reported. Gegen Qinlian Decoction (GQD) is a formulation derived from a classic formula described in the* Treatise on Exogenous Febrile Diseases* over 1000 years ago. GQD formula contains Radix Puerariae,* Coptis chinensis*, Radix Scutellariae, and Radix Liquiritiae. Previous studies have shown that the herbs in GQD could regulate glucose metabolism and its mechanism of action was potentially linked to the improvement of gut microbiota [14]. Antidiabetic effects of GQD formula have been shown in type 2 diabetic rats [15]. We performed this long-term retrospective study to evaluate whether GQD could enhance glycemic control and islet cell function in patients with T2DM whose diabetes were poorly managed.

## 2. Methods

### 2.1. Study Design

Study subjects were recruited from outpatients of endocrine department, Guang'anmen Hospital, China Academy of Chinese Medical Sciences, between July 2009 and June 2014.

### 2.2. Study Population

Outpatients who met the following criteria were eligible for this study: the early type 2 diabetic status of these patients being confirmed according to the diagnosis standard issued by the WHO [16]; receiving Chinese herbal decoction on the main purpose of treating T2DM for at least 6 consecutive months; irrespective of age and sex; having HbA1c ≥ 7.0%, the first recorded HbA1c, fasting insulin (FINS), fasting C-peptide (FCP), and other biochemical measurements being considered as the baseline, while keeping records of HbA1c for at least six months; the fact that all the test results should be measured by the same central laboratory; and having stable diet control and programmed daily exercise during the follow-up treatment.

Patient with any of the following conditions was excluded from the study: having complications of diabetes at first treatment; having serious heart, lung, liver, kidney, brain, or other serious complications or those associated with other primary diseases; having diabetic ketoacidosis or serious infections; participating in other clinical trials; and having unstable antidiabetes drugs during medication. Patients who had poor compliance were withdrawn from the study.

The research protocol was approved by the Guang'anmen Hospital Ethics Committee. This research is a retrospective analysis of clinic cases, which focuses on the documental archives of existing clinical patients. The risk to the participants would be no larger than the minimum risk, in that the exemption of informed consent would not cause any adverse effect on the right and health of the participants; the privacy and personal information would also be protected. As we censored, the ethics committee had agreed to exempt the informed consent and approved the launching of this research.

### 2.3. Intervention

The individual treatment was customized according to the patients' physical conditions on the basis of the standard guide [17]; the dosage of medications remained stable during the first month. Subjects were assessed at each month, while appropriate variation was made in each session according to their measurements and symptoms. Chinese herbal medicine was supplied by Guang'anmen Hospital uniformly. The quality of these herbs and decoction preparation was in accordance with the* Pharmacopoeia of the People's Republic of China* (2005). Subjects took herbal decoction 200 mL two times daily before breakfast and dinner. Each patient should maintain stabilized standard diet and exercise during medication.

For subjects that included oral drugs or insulin therapy throughout the study, the dosages and categories of these medications should be recorded; any change in adjustment and combination related to other diseases should be also recorded at each visit.

### 2.4. Clinical and Biochemical Measurements

The control rate, which was defined as HbA1c level lower than 7%, and the change in HbA1c values were the primary endpoint [18]. HbA1c was measured using affinity HPLC method (Automatic HA-8160 Analyzer HA-8160, Arkray Factory, Inc., Shiga, Japan). Fingertip blood was collected and FPG was measured using a blood glucose analyzer (ACCU-CHEK Active Meter, Roche Diagnostics, Indianapolis, USA). The 2hPG level was measured after taking a standardized meal (Olympus AU640 Analyzer, Olympus Optical Co., Ltd., Shizuoka, Japan). *β*-cell function was evaluated from venous blood to determine plasma insulin and C-peptide level, using the homeostatic model assessment (HOMA) to quantify HOMA insulin resistance (HOMA-IR) and *β*-cell function (HOMA-*β*) [19].

Blood pressure (BP) was collected at each visit. Total cholesterol (TC), triglycerides (TG), high-density lipoprotein (HDL), and low-density lipoprotein (LDL) were measured by enzymatic methods (Olympus AU640 Analyzer, Olympus Optical Co., Ltd., Shizuoka, Japan).

All the changes in test results and symptoms of each outpatient should be recorded in detail, and we collected the records of the first visit and 6, 12, 18, 24, 36, 48, 60 months from the first visit.

### 2.5. Effectiveness Evaluation

A comparison of HbA1c levels before and after medication treatment was made. HbA1c < 7.0% was set as the standard normal value. Comparisons of glucose, plasma insulin, C-peptide, lipids, and BP levels were made. The changes in adjustments of combination drugs and the symptoms would also be evaluated. Subjects developed with diabetes complications during follow-up would also be collected and recorded immediately.

### 2.6. Safety Evaluation

Vital signs were collected at each visit. Routine blood tests, urine tests, stool tests, ECG, hepatic functions (ALT and AST), and renal function (serum creatinine) were also collected. Adverse events were recorded immediately after being reported.

### 2.7. Statistical Analysis

Statistical analysis was performed via SPSS 19.0 software (SPSS Inc., Chicago, IL, USA). Count data was presented as frequency (proportion). Measurement data was presented as the mean ± SD or SE. Related factors were analyzed using multiple linear regression. Paired *t*-test was used to analyze the data before and after medication treatment. All statistical tests were two-sided tests. *p* < 0.05 was considered statistically significant.

## 3. Results

Between July 2009 and June 2014, a total of 142 subjects were enrolled, the baseline was shown in Table 1. There was one subject who developed diabetes kidney disease (DKD) after 12-month treatment and one developed diabetic peripheral neuropathy (DPN) after 22-month treatment, respectively. Among the 72 outpatients who included antidiabetes drugs, there were 3 subjects who added the dosage of drugs, while 42 kept the original, 8 reduced the dosage, and 19 deactivated the drugs during observation period. According to the records, among the 29 outpatients who included insulin, there was 1 subject who added the dosage of insulin, while 17 kept the original, 7 reduced the dosage, and 4 deactivated the insulin after treatment. There were a total of 19 patients who took Chinese herbal decoction alone and kept glycemic controlling well.

### 3.1. HbA1c Change

**Figure 1:**
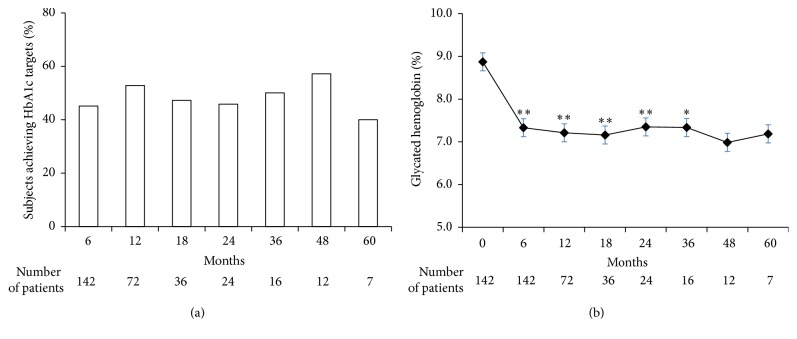
Subjects achieving HbA1c targets (a) and glycated hemoglobin (b) over time. *∗* < 0.05 and *∗∗* < 0.01.

After 6-month treatment, The HbAlc decreased by 1.54 ± 0.18%. 45.07% of the subjects had normal HbA1c after treatment, based on the established criteria. At months 12, 18, 24, 36, 48, and 60, the HbA1c decreased by 1.38 ± 0.25%, 1.59 ± 0.34%, 0.86 ± 0.27%, 1.38 ± 0.58%, 2.43 ± 1.27%, and 2.09 ± 1.71%, respectively, and the control rate was 52.78%, 47.22%, 45.83%, 50.00%, 57.14%, and 40.00% (Figure 1).

We also evaluated the relationship of change in last visited HbA1c with the baseline HbA1c, duration of diabetes, BMI, and age measured by multiple linear regression analysis. It showed that the last recorded HbA1c was significantly related to HbA1c baseline (*X*1), duration of diabetes (*X*2), and BMI (*X*3) (*F* = 992.032, *p* = 0.000), and the regression equation was *Y* = 0.165*X*1 + 0.268*X*2 + 0.011*X*3, *R* ^2^ = 0.965 (Table 2).

### 3.2. FPG and 2hPG

**Figure 2:**
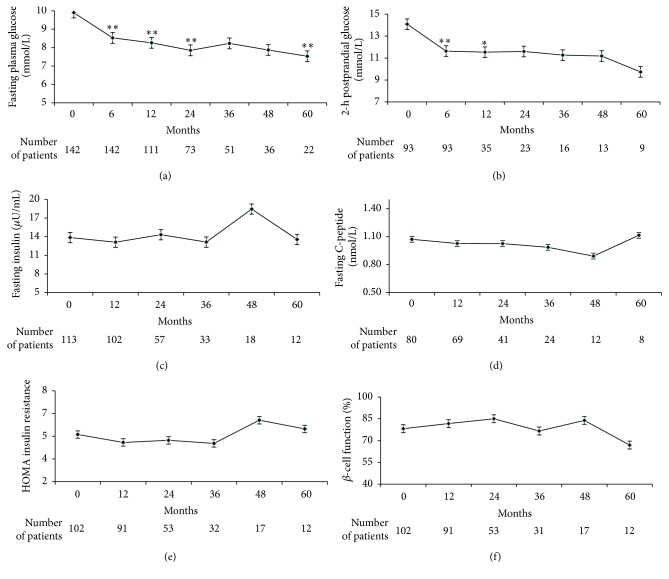
Fasting plasma glucose (a), 2-h postprandial glucose (b), fasting insulin (c), fasting C-peptide (d), HOMA insulin resistance (e), and *β*-cell function (f) over time. *∗* < 0.05 and *∗∗* < 0.01.

A total of 142 subjects measured the FPG for 601 times and 93 subjects measured the 2hPG for 294 times during treatment. Compared to the baseline, the last recorded FPG and 2hPG decreased by 1.26 ± 0.28 and 2.34 ± 0.49 mmol/L, respectively (*p* < 0.05). FPG decreased significantly (*p* < 0.05) after intervention at 6, 12, 24, and 60 months and 2hPG decreased significantly (*p* < 0.05) after intervention at 6 and 12 months (Figures 2(a) and 2(b)).

### 3.3. HOMA-IR and HOMA-*β*

113 subjects measured the fasting insulin for 335 times and 80 subjects measured the C-peptide for 234 times. Subjects who included the insulin were excluded from analysis of this part. The longest comparison observation was 57 months, the shortest was 6 months, and the average observation was 18.55 months. Compared to the baseline, the changes in FINS, FCP, HOMA-IR, and HOMA-*β* among subjects that were last recorded were −1.19 ± 1.05, 0.03 ± 0.07, −0.65 ± 0.48, and −5.29 ± 7.83, respectively. Changes in FINS, FCP, HOMA-IR, and HOMA-*β* over time were shown in Figures 2(c), 2(d), 2(e), and 2(f).

Stratified analysis was performed according to baseline HOMA-IR. HOMA-IR over 2.69 was regarded as insulin resistance according to Chinese characteristics [20]. There were 70 subjects of pretreatment HOMA-IR > 2.69 (61.95%) and 43 cases of pretreatment HOMA-IR ≤ 2.69 (38.05%).

It was found that the changes in FINS, FCP, HOMA-IR, and HOMA-*β* among subjects of pretreatment HOMA-IR > 2.69 that were last recorded were −3.99 ± 1.43, 0.41 ± 0.08, −1.87 ± 0.68, and −20.42 ± 10.60, respectively, while the changes in FINS, FCP, HOMA-IR, and HOMA-*β* among subjects of pretreatment HOMA-IR ≤ 2.69 that were last recorded were 3.39 ± 1.21, −0.01 ± 0.15, 1.42 ± 0.48, and 20.20 ± 9.97, respectively. Stratified analysis also showed that FINS and HOMA-IR decreased significantly among subjects with pretreatment HOMA-IR > 2.69 and increased significantly among subjects with pretreatment HOMA-IR ≤ 2.69 (*p* < 0.05). The change in HOMA-*β* increased significantly among subjects with pretreatment HOMA-IR ≤ 2.69 after intervention (*p* < 0.05).

### 3.4. Other Biochemical Measurements

**Figure 3:**
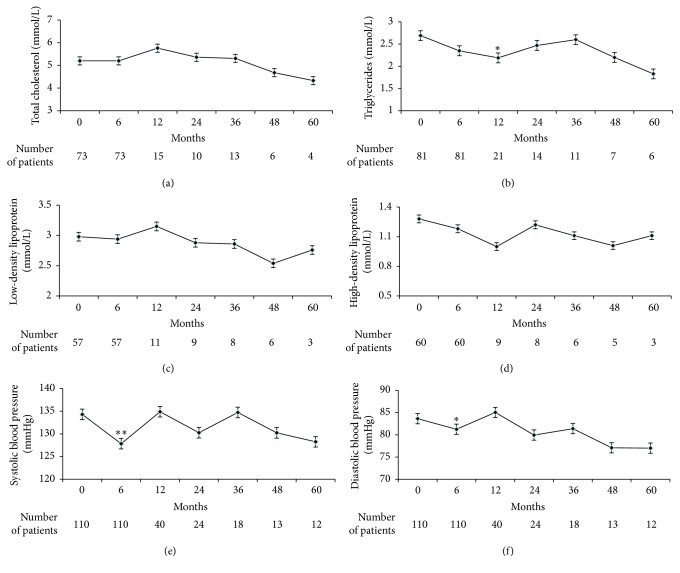
Total cholesterol (a), triglycerides (b), low-density lipoprotein (c), high-density lipoprotein (d), systolic blood pressure (e), and diastolic blood pressure (f) over time. *∗* < 0.05 and *∗∗* < 0.01.

73 subjects measured CHO for 199 times, 81 subjects measured TG for 227 times, 57 subjects measured LDL for 154 times, 60 subjects measured HDL for 154 times, and 110 subjects measured BP for 334 times. Compared with baseline, TG decreased significantly (*p* < 0.05) at 12 months and SBP and DBP decreased significantly (*p* < 0.05) at 6 months (Figure 3).

### 3.5. Safety

There were no serious adverse events that related to Chinese herbal decoction reported during this study. One subject had abnormal liver functions during medication, who was the carrier of hepatitis B virus (HBV). The test results were back to normal after taking hepatoprotectants. Neither hypoglycemia nor cardiovascular event was reported.

## 4. Discussion

Several large-scale clinical trials, such as the United Kingdom Prospective Diabetes Study (UKPDS) and Diabetes Control and Complication Trial (DCCT) [7, 21], have shown that good glycemic control is critical for patients with type 2 diabetes. In the past few decades, the incidence of DM had increased rapidly and the course of disease is not reversible. Thus, prevention of diabetes complications has become the most important part in diabetes management. Though many antidiabetes drugs have emerged in the marketplace, the circumstance is far from satisfactory. Thus, adopting alternative strategies is necessary to improve diabetes treatment, including the use of the Chinese herbal medicine.

Traditional Chinese medicine, which is an important scientific and technological resource with original and independent advantages, has been treating diabetes for thousands of years. The “whole view” and “multitargets” of TCM own unique advantages in controlling complex diseases, such as diabetes. This research is the first long-term observation for evaluating glycemic control and islet cell function treated by Chinese herbal decoction. It provides more detailed assessment of treatment features of herbal decoction and the clinical evidence for rational drug using. It is exciting that our study confirmed that TCM had better glucose controlling and performed protection of islet cell function, which is very important for slowing the disease progression. HbA1c, as the gold standard for evaluating glycemia, has been widely used in the clinic. However, the latest survey in China reported that only 30.15% of 223,114 Chinese T2DM patients had HbA1c lower than 7.0% [9]. Another Chinese study showed that the control rate of HbA1c was 41.1% [22]. It is still not optimistic in the United Kingdom; UKPDS reported about 28%~37% of diabetes patients achieved target levels, that is, HbAlc lower than 7% [23]. Another study reported that 76% of patients had HbAlc > 7.0% [24]. Our study showed that the control rate of HbA1c was between 40.00% and 57.14%. ADOPT showed that the HbA1c was within 7.1%–7.7% in the fifth-year intervention [25]. Our results showed that after five-year intervention HbAlc was 7.2%. Additionally, FPG and 2hPG decreased significantly after intervention. Thus our results preliminarily proved herbal decoction was effective on DM.

The impaired islet cell function is the important pathophysiological mechanism of diabetes, regardless of type I or II diabetes; the progressive exhaustion of islet cell function is the central link of the disease [26]. We could not reverse the diabetes pathology if we are only focusing on the hyperglycemia recovery. It is expected to delay the disease progression for performing early intervention against islet cell dysfunction. Currently the treatment focuses too much on the glycemia while it disregards islet cells as the primary target. The treatment of diabetes in the future should exert more efforts on improving insulin resistance and maintaining normal insulin secretion.

To the best of our knowledge, there is no observation on the efficacy of Chinese herbal decoction treating islet cell function before. Though our information is limited, it is exciting that herbal decoction may improve the islet cell function through assessment. UKPDS has demonstrated that T2DM is a progressive disease that the glycemic deterioration is associated with progressive loss of beta-cell function and no medication could reverse this recession [27]. According to Chinese characteristics, we performed a more rational stratified analysis to evaluate the impact of herbal decoction on islet cell function. It was found that Chinese herbal decoction could mitigate insulin resistance. As for patients without insulin resistance, herbal decoction could increase FINS and islet cell function which may delay the exhaustion of islet function. Moreover, the fifth-year islet cell function maintenance is similar to ADOPT in our study which was 70% to 80% [25]. It is worth mentioning that, in the late period of observation, the islet cell function variation trend was positive. Chinese herbal decoction might alleviate the continuous stimulation of hyperglycemia to islet cell. The effective components may promote the proliferation and differentiation of islet cell, to postpone the exhaustion of islet cell function. This result is encouraging which indicates that traditional Chinese medicine might provide a new alternative therapy for DM.

UKPDS showed that, after six-year intervention, 12.1% of the patients experienced macrovascular events and 5.7% of them experienced microvascular events [27]. In our study the incidences of both macrovascular and microvascular events were low. Traditional Chinese medicine might have advantages in preventing complications. The study showed that Chinese herbal decoction has certain effects on lowering lipids and BP, which reflects the positive impacts of herbal decoction on multiple targets. It is notable that herbal decoction may reduce the dosage or even deactivate the oral drugs or insulin. Safety analysis showed that herbal decoction did not increase the risk of hypoglycemia, which further confirmed the safety of herbal decoction.

Since this study is retrospective, there exist some limitations unavoidably. Large-scale clinical trials or long-term experiment is required to confirm the trends of islet cell function, thus making a more complete assessment and judgment. The study preliminarily proved that Chinese herbal decoction was effective on glycemic control, whereas we could not perform the more detailed stratified analysis as subjects were lost to follow-up during the long-term observation. We also preliminarily proved herbal decoction had positive effect on delaying the disease progression, whereas subjects should measure more timely in the future trials, which is important to explore more deeply on diabetes with complications. Additionally, BMI and waist-to-hip ratio would also be collected in the future study. At present, the precise mechanism of Chinese herbal medicine for glycemia has not been fully understood yet. The suitable scope of application in the corresponding individual syndromes should be further explored and interpreted. To achieve more accurate results and avoid bias, large-scale randomized clinical trials should be launched to estimate the efficacy of herbal decoction on T2DM.

In summary, data from this retrospective study preliminarily demonstrated that the Chinese herbal decoction is effective and safe to use in glycemic controlling and islet cell function improving for diabetes patients. Further clinical studies are needed to confirm the observed effectiveness of Chinese herbal decoction as a new treatment option in the clinical management of diabetes. By the application of TCM comprehensive system for the prevention and control, the prevention of diabetes complication would be improved significantly. The tendency of the rising rates of diabetes and its complications would be controlled, or even the inflection point would appear.

## Figures and Tables

**Table 1:** Characteristics of study subjects at baseline.

	Chinese herbal decoction (*n* = 142)
Age (years)	50.54 ± 12.23
Gender (M/F)	59.86%/40.14%
Height (m)	1.68 ± 0.08
Weight (kg)	72.00 ± 12.56
Duration of diabetes (month)	72.96 ± 63.45
Duration of treatment (month)	21.88 ± 18.05
Times of therapy (*n*)	11.90 ± 7.77
History of hypertension (*n*)	59 (41.55%)
History of dyslipidemia (*n*)	65 (45.77%)
History of fatty liver (*n*)	32 (22.54%)
History of hyperuricemia (*n*)	8 (5.63%)
Combined with insulin (*n*)	29 (20.42%)
Combined with metformin (*n*)	37 (26.06%)
Combined with repaglinide (*n*)	14 (9.86%)
Combined with gliclazide (*n*)	8 (5.63%)
Combined with glimepiride (*n*)	5 (3.52%)
Combined with acarbose (*n*)	24 (16.90%)
Combined with rosiglitazone (*n*)	4 (2.82%)
HbA1c (%)	8.98 ± 2.02
FPG (mmol/L)	9.96 ± 2.94
2hPG (mmol/L)	14.07 ± 4.90
CHO (mmol/L)	5.43 ± 1.19
TG (mmol/L)	2.71 ± 2.72
LDL (mmol/L)	3.17 ± 0.97
HDL (mmol/L)	1.26 ± 0.43
Systolic pressure (mmHg)	134.71 ± 20.51
Diastolic pressure (mmHg)	83.94 ± 9.68
UA (*μ*mol/L)	315.81 ± 118.73
ALT (U)	37.42 ± 47.11
AST (U)	25.58 ± 14.75

Values are expressed as mean ± SD.

**Table 2:** Factors influencing HbA1c control of patients.

	*B*	SE	Standardized regression coefficients	*T*	*p*
HbA1c at baseline	0.165	0.020	0.562	8.226	0.000
Duration of diabetes	0.268	0.054	0.328	4.978	0.000
BMI	0.011	0.002	0.130	4.783	0.000
Age	0.113	1.583	0.116	0.151	0.063
