# Corrigendum to “The Efficacy and Safety of Chinese Herbal Decoction in Type 2 Diabetes: A 5-Year Retrospective Study”

**DOI:** 10.1155/2017/7683754

**Published:** 2017-02-15

**Authors:** Jiaxing Tian, Fengmei Lian, Xiaotong Yu, Yashan Cui, Tianyu Zhao, Yang Cao, Xiaolin Tong

**Affiliations:** ^1^Beijing University of Chinese Medicine, Beijing 100029, China; ^2^Guang'anmen Hospital, China Academy of Chinese Medical Sciences, Beijing 100053, China; ^3^Xiyuan Hospital, China Academy of Chinese Medical Sciences, Beijing 100091, China; ^4^First Teaching Hospital, Tianjin University of Traditional Chinese Medicine, Tianjin 300192, China

In the article titled “The Efficacy and Safety of Chinese Herbal Decoction in Type 2 Diabetes: A 5-Year Retrospective Study” [1], there was an error in the order of the first and second affiliations. The corrected affiliation list is shown above.
